# Evaluation of CYP1A2 activity: Relationship between the endogenous urinary 6‐hydroxymelatonin to melatonin ratio and paraxanthine to caffeine ratio in dried blood spots

**DOI:** 10.1111/cts.13263

**Published:** 2022-03-26

**Authors:** Gaëlle Magliocco, Jules Desmeules, Caroline Flora Samer, Aurélien Thomas, Youssef Daali

**Affiliations:** ^1^ Division of Clinical Pharmacology and Toxicology Geneva University Hospitals Geneva Switzerland; ^2^ School of Pharmaceutical Sciences University of Geneva Geneva Switzerland; ^3^ Institute of Pharmaceutical Sciences of Western Switzerland University of Geneva Geneva Switzerland; ^4^ Faculty of Medicine University of Geneva Geneva Switzerland; ^5^ Forensic Toxicology and Chemistry Unit CURML, Lausanne University Hospital, Geneva University Hospitals Lausanne, Geneva Switzerland; ^6^ Faculty Unit of Toxicology, Faculty of Biology and Medicine CURML, University of Lausanne Lausanne Switzerland

## Abstract

The suitability of the endogenous 6‐hydroxymelatonin/melatonin urinary metabolic ratio as a surrogate for the paraxanthine/caffeine ratio to predict cytochrome P450 1A2 (CYP1A2) activity was assessed in this study. Twelve healthy volunteers completed four study sessions spread over 1 month (including overnight urine collection with first morning voids collected separately). Except for the third session, volunteers were asked to abstain from methylxanthine‐containing beverages and foods at least 24 h before urine collection. At the end of urine collection, subjects were given a caffeinated beverage and capillary blood samples were collected 2 h after the drink administration. A significant linear relationship between the 6‐hydroxymelatonin/melatonin ratios from 12‐h urine samples and first morning voids was observed (*R* ^2^ = 0.876, *p* < 0.0001). In contrast to the paraxanthine/caffeine ratio, consumption of methylxanthine‐containing beverages during session three did not significantly influence the 6‐hydroxymelatonin/melatonin ratios compared with the other sessions requiring abstinence from caffeine. A larger intra‐ and interindividual variability in the 6‐hydroxymelatonin/melatonin ratios compared with the paraxanthine/caffeine ratio was also observed. A very weak correlation was observed between the paraxanthine/caffeine ratio and both of the endogenous 6‐hydroxymelatonin/melatonin ratios (Pearson *r* < 0.35, *p* < 0.05). All these results question whether this endogenous metric could adequately reflect CYP1A2 activity or substitute for the probe caffeine. Additional studies with larger study samples are needed to examine this endogenous metric in more details.

**Study Highlights**

**WHAT IS THE CURRENT KNOWLEDGE ON THE TOPIC?**

The use of endogenous markers offers numerous advantages in terms of safety and convenience over exogenous probes to phenotype cytochrome P450 (CYP450) activity. Melatonin, a hormone endogenously synthetized by the pineal gland at night, is essentially hydroxylated into 6‐hydroxymelatonin by CYP1A2. Previous studies have shown successful use of exogenous melatonin for CYP1A2 phenotyping, prompting interest in possible CYP1A2 phenotyping via endogenous 6‐hydroxymelatonin to melatonin metabolic ratio.

**WHAT QUESTION DID THIS STUDY ADDRESS?**

This study assessed the suitability of the endogenous 6‐hydroxymelatonin to melatonin urinary metabolic ratio to substitute caffeine to predict CYP1A2 activity.

**WHAT DOES THIS STUDY ADD TO OUR KNOWLEDGE?**

The endogenous 6‐hydroxmelatonin to melatonin ratio could be measured noninvasively in morning spot urine without substantial impact of caffeine intake. However, despite a weak positive correlation with the paraxanthine to caffeine ratio, current results are insufficient to affirm the ability of the 6‐hydroxmelatonin to melatonin ratio to adequately reflect CYP1A2 activity, or even to outperform the probe caffeine.

**HOW MIGHT THIS CHANGE CLINICAL PHARMACOLOGY OR TRANSLATIONAL SCIENCE?**

The use of the endogenous 6‐hydroxymelatonin to melatonin ratio measured in morning spot urine represents an interesting starting point for larger clinical studies evaluating this endogenous metric.

## INTRODUCTION

Cytochrome P450 1A2 (CYP1A2) is one the most important CYP enzymes in the human liver.^1^ Its expression has been reported to vary from 40‐ to 130‐fold in the human population.^2^ The causes of variability in CYP1A2 activity are numerous and primarily include non‐genetic factors, such as smoking, drug–drug interactions, age, or gender.^1^ Globally, clinical impact of CYP1A2 genotyping is prone to conflicting evidence in the literature, and phenotype prediction from CYP1A2 genotype is not well‐established.^3^, ^4^, ^5^ CY1A2 phenotyping thus appears as the best tool to characterize enzyme function, representing a major step toward personalized medicine. Caffeine is the most commonly used probe to phenotype CYP1A2 activity.^6^, ^7^ Caffeine metabolism goes through several CYP450 enzymes, but ~ 95% of a total caffeine dose is metabolized by CYP1A2. Its main metabolites are theobromine (about 10%) and paraxanthine (about 80%) through N3‐demethylation.^6^, ^7^ Prior to CYP1A2 phenotyping using caffeine, study participants or patients are usually asked to refrain from methylxanthine‐containing foods and beverages, which constitutes a major drawback of this procedure.^7^

In the field of personalized medicine, there is considerable interest in endogenous compounds metabolized through drug‐metabolizing enzymes.^8^, ^9^ Indeed, CYP450 enzymes are subject to high intra‐ and interindividual variability, requiring effective and nonburdensome tools to characterize their activity in a given subject at a specific time.^10^ The use of endogenous markers offers numerous advantages over exogenous probes to phenotype CYP450 activity. It is a very safe approach because there is no need to ingest or inject xenobiotics, eliminating any risk of allergy, intolerance or adverse effects.^8^ Moreover, phenotyping using endobiotics could help provide knowledge of CYP450 activity in populations where the administration of exogenous compounds may be inconvenient and unethical, such as pregnant women, children, or the elderly.^8^, ^11^, ^12^ In this context, melatonin is a hormone synthetized by the pineal gland at night from serotonin by a two step‐process.^13^ Its metabolism is essentially mediated by CYP1A2, which catalyzes hydroxylation of melatonin into 6‐hydroxymelatonin.^14^ In 12 healthy volunteers who received a single oral dose of 25 mg of melatonin and 100 mg of caffeine, melatonin clearance correlated significantly with caffeine clearance (*r*_*s*_ = 0.748; *p* = 0.005), suggesting that melatonin may be an alternative to caffeine as a probe drug for CYP1A2 phenotyping.^15^ However, measurement of endogenous melatonin and its metabolite, 6‐hydroxymelatonin, has been poorly explored as a marker of CYP1A2 activity. Von Bahr et al.^16^ measured endogenous melatonin levels in serum and urine from seven healthy volunteers. Following administration of fluvoxamine 50 mg, a potent CYP1A2 inhibitor, they observed an increase in the area under the concentration‐time curve (AUC) of melatonin by 2.8‐fold and in urinary melatonin excretion over 14 h by 2.1‐fold compared with placebo (*p* < 0.05).^16^, ^17^ Likewise, Skene et al.^18^ showed that in addition to increasing AUC of melatonin by a factor of 2.9 (*p* < 0.01), fluvoxamine 100 mg decreased urinary excretion of 6‐sulphatoxymelatonin, the conjugated form of 6‐hydroxymelatonin, between midnight and 9 a.m. compared to the control session in eight study participants (*p* < 0.01).

The objective of this study was to provide additional data concerning the 6‐hydroxymelatonin to melatonin metabolic ratio in human urine. In particular, we investigated whether overnight urine (collected from 9 p.m. to 9 a.m.) could be substituted by first morning void samples for the purpose of CYP1A2 phenotyping. The use of the paraxanthine/caffeine ratio in dried blood spots (DBS) in a single‐time point drawn 2 h after caffeine administration, is a validated approach developed by Bosilkovska et al. to determine CYP1A2 activity.^19^, ^20^ Therefore, correlations between the metabolic ratio of 6‐hydroxymelatonin/melatonin in urine and paraxanthine/caffeine in DBS were also assessed. Finally, the intra‐ and interindividual variabilities of the 6‐hydroxymelatonin/melatonin ratio, as well as the impact of caffeine intake on this endogenous marker were established.

## METHODS

### Study design and population

The study protocol (NCT04420611) was approved by the Geneva Research Ethics Committee and the study was conducted according to the guidelines of the Declaration of Helsinki. All participants provided written informed consent before inclusion. Participants unable to abstain from alcohol or methylxanthine‐containing beverages and food for 24 h, sensitive to coffee/cola beverages, or taking irregularly (i.e., non‐daily or inconstant dosages) drugs, and foods or tobacco‐derived products that modulate CYP1A2 activity were excluded.

**FIGURE 1:**
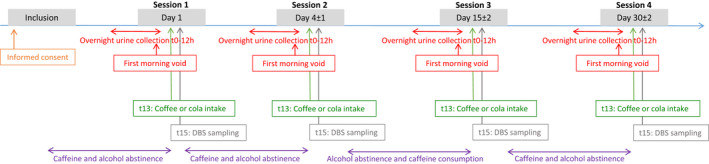
Study design. DBS, dried blood spot

The study was conducted in four sessions spread over 1 month, as described in Figure 1.

During each study session, overnight urine (including urine voided during the night) were collected for 12 h (from 9 p.m. to 9 a.m.). First morning voids were collected separately in order to obtain an aliquot analyzed independently from the 12 h collection. The remaining urine from the first morning voids’ container was combined in the 12‐h urine bottle to create the 12‐h sample.

Volunteers were asked to refrain from alcohol consumption and methylxanthine‐containing beverages and foods at least 24 h before the urine collection, except for session three regarding the consumption of methylxanthines. Before this second‐to‐last session, subjects were on an unrestricted caffeine intake during the 24 h preceding urine collection. For subjects who did not drink caffeinated beverages daily, they were asked to consume at least one cup of coffee or cola 24 h prior to overnight urine collection to ensure caffeine intake. At around 9 a.m., after each overnight urine collection, subjects were administered a cup of coffee or cola (depending on individual preference) after an overnight fast. Capillary blood samples from a small finger prick (10 μL) were collected 2 h after the beverage was given. The measurement of the paraxanthine/caffeine ratio in DBS 2 h after the administration of caffeine is based on a previous report by Bosilkovska et al.^20^ This work showed a significant correlation between two ratios of paraxanthine/caffeine (i.e., one using the area under the concentration‐time curve from time zero to the last quantifiable concentration [AUC_last_] in plasma and the other using a single time point capillary DBS at 2 h), under basal condition as well as after the intake of inducer/inhibitors. Capillary whole blood was collected using microsampling HemaXis DB10 kits purchased from DBS System SA, allowing collection of controlled volume (10.0 ± 0.5 μL per spot; Gland, Switzerland). In addition, Bosilkovska et al.^19^ observed strong linear relationships between caffeine and paraxanthine concentrations in capillary DBS and venous plasma, as indicated by high values of coefficients of determination (*R* ^2^ ranging from 0.843–0.985), suggesting that DBS may be an alternative sampling technique to conventional venous plasma collection.

### Quantification of 6‐hydroxymelatonin/melatonin, and paraxanthine/caffeine

All the compounds were quantified in urine or DBS using an Agilent 1290 Infinity series LC system from Agilent (Paolo Alto, CA) coupled to a 6500 QTtrap triple quadrupole linear ion trap mass spectrometer from AB Sciex equipped with an electrospray ionization (Darmstadt, Germany). Both methods were validated in terms of selectivity, accuracy, precision, recovery, and matrix effect.

Urinary 6‐hydroxymelatonin and melatonin were quantified according to the method described by Magliocco et al.^21^ using deuterated analogues as internal standards. Very briefly, following solid phase extraction and enzymatic hydrolysis, quantification was performed using a single reverse‐phase high‐performance liquid chromatography coupled with tandem mass spectrometry (LC‐MS/MS) method. Because 6‐hydroxymelatonin is largely conjugated into 6‐sulfatoxymelatonin and 6‐hydroxymelatonin glucuronide, we used β‐glucuronidase/arylsulfatase solution from *Helix pomatia* for rapid hydrolysis of the glucuronide and sulfate linkage.^21^

The analyses of paraxanthine and caffeine in DBS were performed using the LC‐MS/MS method, as described in the study of Bosilkosvka et al.^19^ using methanol as extraction solvent.

### Statistical analysis

Linear regression and Bland–Altman plot were used to evaluate the relationship between the 6‐hydroxymelatonin/melatonin ratio from overnight urine samples and first morning voids. For the Bland–Altman analysis, the average of the ratios in 12‐h (overnight) and first morning voids urine samples at each session were measured and the percentage of difference was calculated as follows:

$$\frac{\frac{\text{Hydroxymelatonin}}{\text{Melatonin}}\text{in first morning voids}-\frac{\text{Hydroxymelatonin}}{\text{Melatonin}}\;\text{in overnight urine samples}}{\text{Average}\;}\cdot 100$$

We reported the −20 to +20% range in the Bland–Altman plots, because at least 67% of the observations must fall within this range for two methods to be considered as providing similar results according to the European Medicines Evaluation Agency (EMEA) guidelines (cross‐validation acceptance criteria) and previous reports.^22^, ^23^, ^24^ In addition, based on the US Food and Drug Administration (FDA) Bioequivalence Hearing, a difference of 20% is not considered clinically significant.^25^

Normality was tested using the Kolmogorov–Smirnov test and significant outliers were identified by the ROUT method and removed (*Q* = 1%). The effect of caffeine on the 6‐hydroxymelatonin/melatonin and paraxanthine/caffeine ratios was assessed via one‐way analysis of variance (ANOVA) followed by Tukey’s post hoc tests. Pearson’s correlation coefficients were calculated to measure the correlation between the 6‐hydroxymelatonin/melatonin and paraxanthine/caffeine ratios.

All statistical analyses were performed using GraphPad Prism 8.0.1 software (San Diego, CA). A *p* value less than or equal to 0.05 was considered statistically significant.

## RESULTS

### Subjects

A total of 12 healthy volunteers (6 men and 6 women) were recruited. The median age was 29 years (range 24–61 years). All subjects were White. One subject was a light daily smoker (1–5 cigarettes/day). Two female volunteers took oral contraceptive pills but no study session was scheduled during the 7‐day contraceptive‐free interval. The other volunteers were not taking any other drugs modulating CYP1A2 activity.

Using the ROUT method with a preselected false‐discovery rate of 1%, data from one volunteer in session one were removed from the data analysis because of extreme differences in the 6‐hydroxymelatonin/melatonin ratios compared with other sessions and other volunteers. Normal distributions of the data were confirmed by the Kolmogorov–Smirnov test.

### Overnight versus first morning void urine samples

On average, urine volume was 2.3‐fold higher (range 1.1–7.6) during the 12‐h collection compared with samples from the first morning voiding.

**FIGURE 2:**
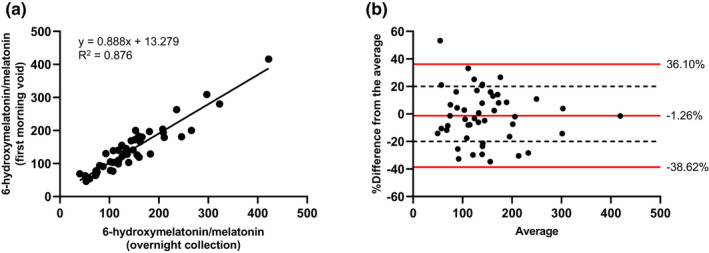
(a) Linear regression between 6‐hydroxymelatonin/melatonin ratios measured in 12‐h (overnight) and first morning voids urine samples. (b) Bland–Altman comparison of the 6‐hydroxymelatonin/melatonin ratio in 12‐h (overnight) and first morning voids urine samples. This plot illustrates the average of the ratios measured in 12‐h (overnight) and first morning voids urine samples at each session (*x*‐axis) versus the difference between the ratios in first morning voids and 12‐h (overnight) urine samples (expressed as a percentage of the average, *y*‐axis). The mean bias and 95% limits of agreement are represented as solid, red lines, whereas the dashed lines show the −20 to +20% range

Figure 2a illustrates the relationship between the 6‐hydroxymelatonin/melatonin urinary metabolic ratio measured from 12‐h urine samples and first morning voids in 12 participants at all four study sessions. A significant linear relationship was measured between these two different urine collection methods. The Bland–Altman plot (Figure 2b) shows that the bias (average of the differences) between the 12‐h urine samples and the first morning voids is around −1.26%. The limits of agreement of the Bland–Altman plot (calculated as bias ±1.96 times the standard deviation of the relative difference) were between −38.62% and 36.10%. At least 67% of the values were within the ±20% threshold set in the EMEA guidelines (cross‐validation acceptance criteria).

### Influence of caffeine intake

**FIGURE 3:**
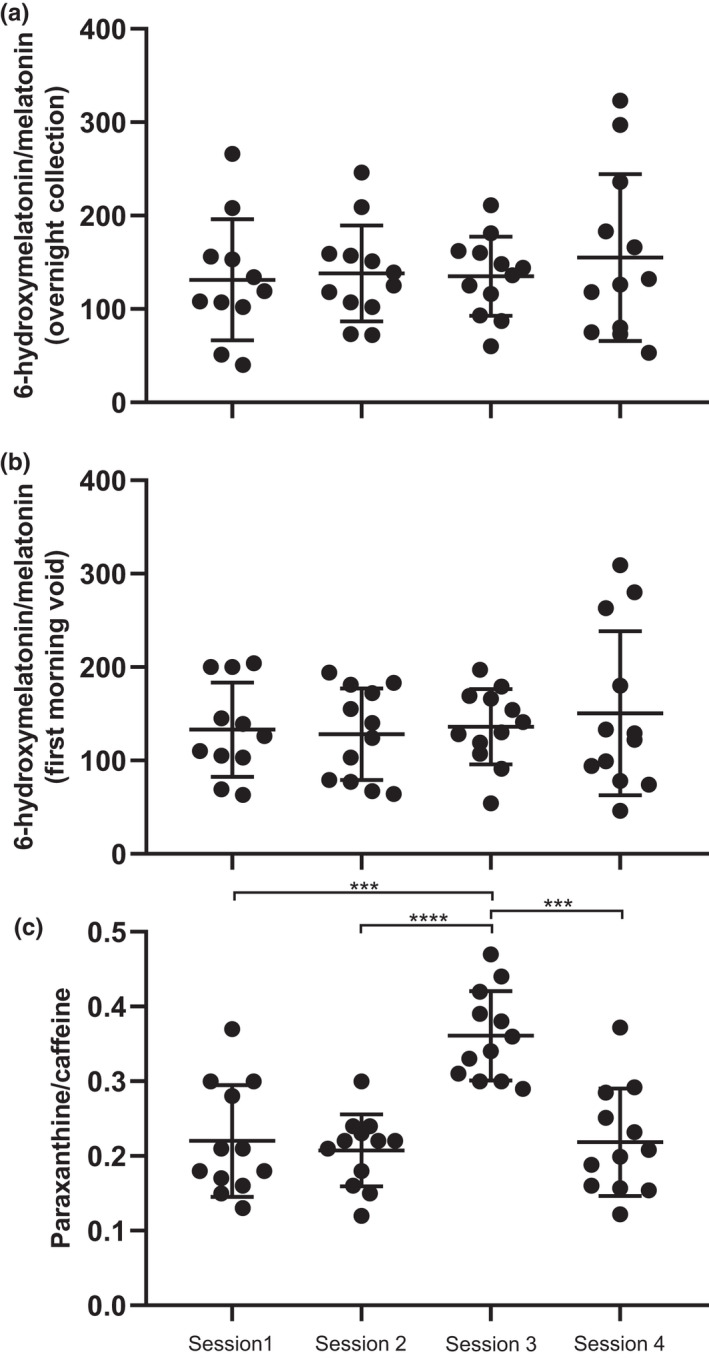
Scatter dot plots with horizontal lines representing means ± SD. (a) Distribution of the endogenous 6‐hydroxymelatonin/melatonin ratio measured in 12‐h (overnight) urine samples. (b) Distribution of the endogenous 6‐hydroxymelatonin/melatonin ratio measured in first morning voids urine samples. (c) Distribution of the paraxanthine/caffeine ratio measured in dried blood spot samples. Sessions one, two, and four represent baseline conditions, including abstention from alcohol and caffeine at least 24 h before the urine collection. In contrast, in session three, subjects were asked to consume at least one cup of coffee or cola during this 24‐h period. *** *p* < 0.001; **** *p* < 0.0001

As illustrated in Figure 3a,b, the mean 6‐hydroxymelatonin/melatonin ratios in 12‐h (overnight) and first morning voids urine samples were not significantly different among the four study sessions, including the third session.

The following paraxanthine/caffeine metabolic ratios were measured in DBS samples in sessions one, two, three, and four: 0.22 ± 0.07, 0.21 ± 0.05, 0.36 ± 0.06, and 0.22 ± 0.07, respectively (Figure 3c). The paraxanthine/caffeine ratio was significantly increased by ~ 1.6‐fold when subjects were requested to consume methylxanthine‐containing beverages and foods (session 3) compared with the other sessions (sessions 1, 2, and 4). Examining the individual data (Appendix S1), the paraxanthine/caffeine metabolic ratio was increased in all subjects during session three compared with the other sessions, except for one participant whose ratio remained rather stable.

### Intra‐ and interindividual variability

In our study, the mean intersubject coefficients of variation (CVs) of the 6‐hydroxymelatonin/melatonin ratios measured in first morning voids and 12‐h (overnight) urine samples were 41.0 ± 12.2% and 43.9 ± 11.9%, respectively (Appendix S1). The mean intersubject CV of the paraxanthine/caffeine ratio in DBS at 2 h was 26.7% ± 8.3%.

The mean intrasubject CVs of the paraxanthine/caffeine ratio in DBS at 2 h and 6‐hydroxymelatonin/melatonin ratios measured in first morning voids and 12‐h (overnight) urine samples are described in Table 1. Because of the significant impact of uncontrolled caffeine consumption on the paraxanthine/caffeine ratio, session three was excluded for assessment of intra‐individual variability in this metric (see section Influence on caffeine intake). The light smoker, corresponding to the subject number seven, showed globally higher 6‐hydroxymelatonin/melatonin urinary ratios (first morning voids and overnight collection) than other individuals, except for the participant number eight. However, such results were not reflected in the paraxanthine/caffeine ratio.

**TABLE 1:** Intra‐individual variability of the endogenous 6‐hydroxymelatonin/melatonin ratios measured in first morning voids and 12‐h (overnight) urine samples, as well as the paraxanthine/caffeine ratio measured in dried blood spot samples at 2 h from three different sessions (session 1, 2, and 4)

Subject	6‐hydroxymelatonin/melatonin (first morning voids)		6‐hydroxymelatonin/melatonin (overnight samples)		Paraxanthine/caffeine (DBS at 2 h)	
	Mean ± SD	CV (%)	Mean ± SD	CV (%)	Mean ± SD	CV (%)
1	163 ± 34.2	21.0	137 ± 14.7	10.8	0.25 ± 0.04	15.2
2	87.1 ± 15.1	17.3	92.7 ± 16.7	18.9	0.20 ± 0.02	7.7
3	114 ± 21.7	19.1	121 ± 15.9	13.0	0.16 ± 0.01	6.9
4	116 ± 11.7	10.0	130 ± 25.0	19.3	0.25 ± 0.05	17.7
5	67.0 ± 6.2	9.3	65.8 ± 12.9	19.6	0.20 ± 0.03	16.9
6	60.6 ± 12.7	21.0	55.2 ± 16.6	30.1	0.20 ± 0.04	19.7
7	217 ± 64.3	29.6	194 ± 60.4	31.2	0.19 ± 0.08	44.3
8	212 ± 85.8	40.5	226 ± 83.4	36.9	0.26 ± 0.04	14.5
9	161 ± 106	65.7	195 ± 113	58.0	0.35 ± 0.04	11.6
10	115 ± 23.2	20.3	106 ± 22.3	21.1	0.13 ± 0.01	10.7
11	175 ± 39.3	22.5	219 ± 42.4	19.4	0.17 ± 0.02	10.8
12	189 ± 12.9	6.9	178 ± 26.7	15.0	0.21 ± 0.03	13.8
All subjects	137 ± 64.2	23.6 ± 16.1	142 ± 69.2	24.4 ± 13.1	0.22 ± 0.06	15.8 ± 9.8

Abbreviations: CV, coefficient of variation; DBS, dried blood spot.

### Correlation between the DBS paraxanthine/caffeine ratio and the endogenous urinary 6‐hydroxymelatonin/melatonin ratio

**FIGURE 4:**
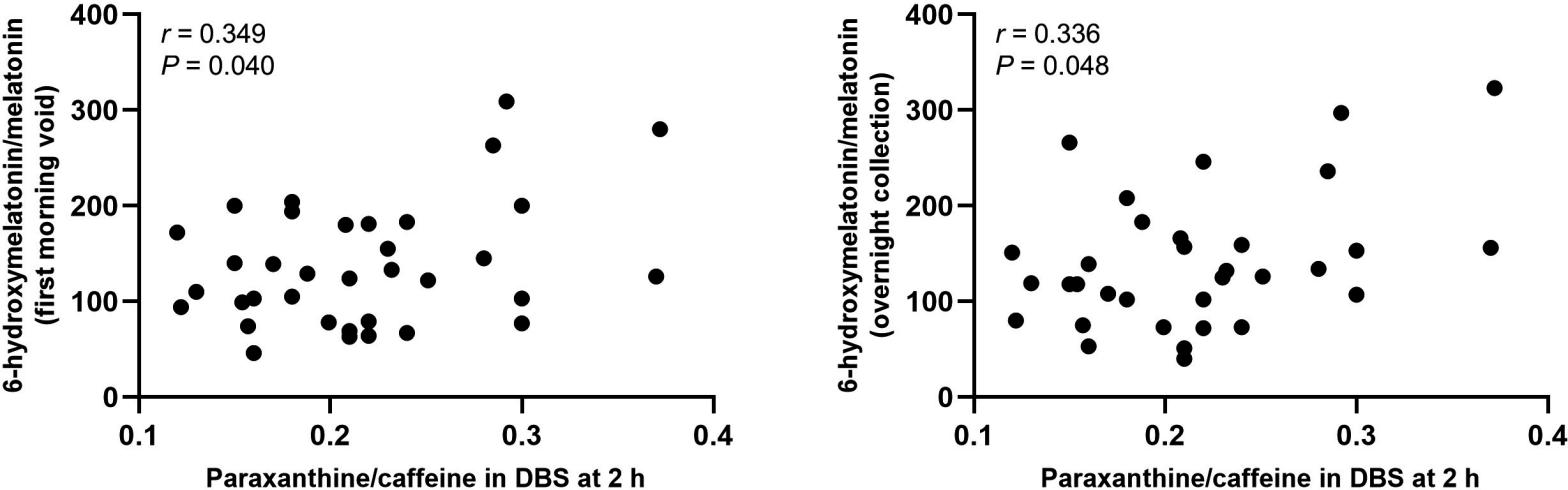
Pearson’s correlation of paraxanthine/caffeine ratio in dried blood spot with the 6‐hydroxymelatonin/melatonin urinary ratios (first morning voids and overnight collection) at study sessions one, two, and four (caffeine abstinence)

Figure 4a,b show the results regarding the Pearson’s correlation coefficients between the 6‐hydroxymelatonin/melatonin urinary ratios (first morning voids and overnight collection) and the paraxanthine/caffeine DBS ratio at 2 h for each subject at sessions one, two, and four (sessions measured combined). Because of the significant influence of caffeine consumption on the paraxanthine/caffeine ratio in DBS (see section Influence on caffeine intake), session three was not considered in the correlation assessments.

## DISCUSSION

Melatonin is secreted by the pineal gland to regulate, primarily, circadian rhythms and sleep through melatonin receptors.^26^ Melatonin concentrations start to increase at around 9 p.m., reaching maximum plasma concentration between 3 and 4 a.m.^27^ In agreement with this, previous reports have shown that melatonin and its conjugated metabolite 6‐sulfatoxymelatonin have maximum urinary excretion during the night, remaining elevated until about 9 a.m.^28^ In order to develop a method of urine collection that is the least burdensome, we compared the 6‐hydroxymelatonin/melatonin urinary ratios measured in urine samples collected strictly from 9 p.m. to 9 a.m. versus uncontrolled first morning void (consisting of ~ 2 to 10 h of urine production, depending on bedtime, time of awakening, and sleep interruption). Despite large differences in the amounts of volume collected between the two types of collection (2.3 times greater volume in the 12‐h urine samples than in the first morning voids), we showed a significant linear relationship between the 6‐hydroxymelatonin/melatonin ratios estimated either from first morning voids or from 12‐h urine sample collection (from 9 p.m. to 9 a.m.). These results suggest that a shorter, more clinically manageable, early morning spot urine collection may replace the more controlled and burdensome 12‐h method. It is likely that measuring the metabolic ratio (e.g., metabolite/substrate) minimizes variability caused by the circadian rhythm. Similarly, the 6β‐hydroxycortisol/cortisol urinary metabolic ratio remains constant throughout the day in spite of the circadian variations.^29^, ^30^

In the third session, subjects followed an unrestricted caffeine diet. Participants who did not drink caffeinated beverages daily were asked to consume at least one cup of coffee or cola 24 h before overnight urine collection to ensure caffeine intake. No effect of caffeine was observed on the endogenous 6‐hydroxymelatonin/melatonin urinary metabolic ratio. Therefore, it appears that routine consumption of caffeinated beverages can be maintained before measurement of this metric. No impact of caffeine was also noted when examining melatonin and 6‐hydroxymelatonin excretion rates measured separately (data not shown). These results corroborate the findings of Härtter et al.^31^ that observed no significant effect of caffeine ingestion 12 or 24 h before oral melatonin intake (6 mg) on melatonin concentration at 1.5 h. Despite discrepancies, previous studies reported important effects of caffeine on melatonin pharmacokinetics. For instance, Ursing et al.^32^ demonstrated a significant increase in the AUC of endogenous melatonin (measured from 10 p.m. to 8 a.m.) after 12 healthy volunteers received 200 mg of caffeine in the evening (at 10 p.m.) compared to a placebo. They hypothesized that caffeine could promote melatonin release from pinealocytes or possible effect of caffeine on CYP1A2. Wright et al.^33^ observed the opposite: 200 mg of caffeine, ingested twice a night, decreased nocturnal salivary melatonin levels. They suggested that caffeine may influence melatonin levels through action on adenosine A_2b_ receptors in the pineal gland. Further studies are therefore needed to confirm the absence of effect of caffeine on the endogenous 6‐hydroxymelatonin/melatonin urinary metabolic ratio.

A 24‐ or 48‐h abstinence from caffeine consumption before phenotyping of CYP1A2 activity using caffeine as a probe is often required and highly burdensome for patients and study participants.^7^, ^34^, ^35^ In our study, the paraxanthine/caffeine ratio in DBS at 2 h was significantly higher when methylxanthine‐containing foods and beverages were allowed (session 3) compared with the abstinence phases (sessions 1, 2, and 4). Similar results have been reported in other studies.^36^, ^37^ Caffeine and paraxanthine have different pharmacokinetic profiles. It has been demonstrated that 8 to 10 h after caffeine ingestion, paraxanthine levels exceed those of caffeine in plasma.^20^, ^38^ Despite the paucity of studies on the kinetics of caffeine and paraxanthine in humans after daily caffeine intake, we found that half‐life of caffeine (~ 4.3 h) is shorter than the half‐life of paraxanthine (~ 7.8 h), which may explain the higher paraxanthine/caffeine ratio observed in session three compared with sessions one, two, and four of the present study.^39^ Previous studies also reported the possible quantification of dietary caffeine in urine or saliva to measure CYP1A2 activity.^40^, ^41^, ^42^ This interesting approach needs to be further explored, especially with regard to the nonlinear (dose‐dependent) pharmacokinetics of caffeine and possible saturation of CYP1A2‐mediated metabolism.^43^ Indeed, given the large differences observed in our studies between sessions with and without caffeine abstinence, we question whether it is possible to phenotype CYP1A2 activity through the paraxanthine/caffeine ratio measured in random samples and/or uncontrolled conditions (i.e., unrestricted caffeine intake). Additional studies are needed to compare CYP1A2 phenotyping under a standardized sampling method versus randomly collected samples, and time‐controlled caffeine administration versus no prior caffeine abstinence.

In this particular study, intra‐individual variation in the paraxanthine/caffeine ratio in DBS at 2 h when comparing ratios at sessions one, two, and four (over a period of ~ 1 month) was 15.8 ± 9.8%. Measured over a 12‐week period, the plasma paraxanthine/caffeine ratio at 5 h showed a similar mean intrasubject CV of 17.6 ± 6.0% and 16.2 ± 5.9% in young (*n* = 16) and elderly (*n* = 16), respectively, in a study by Simon et al.^44^ In contrast, intra‐individual variability in the 6‐hydroxymelatonin/melatonin ratios measured in first morning voids and 12‐h (overnight) urine samples was slightly higher (mean CV 23.6 ± 16.1% and 24.4% ± 13.1, respectively). It is likely that uncontrolled factors are affecting urinary excretion rates of endogenous melatonin and 6‐hydroxymelatonin. If uncontrolled factors affect the 6‐hydroxymelatonin/melatonin ratios resulting in higher intra‐individual variability than the plasma paraxanthine/caffeine ratio, it seems clear that this endogenous metric is less likely to reliably capture CYP1A2 activity.

In this study, the urinary 6‐hydroxymelatonin/melatonin ratios estimated either from first morning voids or from 12‐h urine sample collection (from 9 p.m. to 9 a.m.) also exhibited higher interindividual variability than the paraxanthine/caffeine ratio with a mean intersubject CV estimated at 41.0 ± 12.2% and 43.9 ± 11.9%, respectively. The mean intersubject CV of the paraxanthine/caffeine ratio in DBS at 2 h was 26.7 ± 8.3%. Previous research work reported higher intersubject CV for plasma paraxanthine/caffeine ratios measured at a single time point (ranging from ~ 29 to 48%).^44^, ^45^, ^46^ Because of the small sample size, we did not investigate potential factors that may contribute to interindividual variability. However, it seems essential to assess whether the 6‐hydroxymelatonin/melatonin ratio is still able to capture the impact of factors contributing significantly to CYP1A2 modulation, despite slightly higher intra‐ and interindividual variability than the paraxanthine/caffeine ratio. In particular, the impact of gender, genetics, pathological states (e.g., liver disease), or comedication on the urinary 6‐hydroxymelatonin/melatonin ratio should be further investigated in future projects, taking into account the variability measured in this study to estimate the sample size.^1^, ^47^, ^48^, ^49^ In particular, smoking, a potent inducer of CYP1A2 enzyme activity, is expected to increase any metabolite‐to‐substrate ratio. In this study, only one subject reported being a light smoker, which was possibly reflected in the urinary 6‐hydroxymelatonin/melatonin ratio in first morning voids but not the paraxanthine/caffeine ratio. It also seems important to measure whether different sleep patterns (e.g., early risers vs. late sleepers) would have an impact on the 6‐hydroxymelatonin/melatonin ratio.

Caffeine is usually considered the reference probe for CYP1A2 because its metabolism is primarily mediated by CYP1A2. In this study, the relationship between the endogenous 6‐hydroxymelatonin/melatonin urinary ratio and the paraxanthine/caffeine ratio in DBS at 2 h was examined. We observed significant correlations between paraxanthine/caffeine ratio and endogenous 6‐hydroxymelatonin/melatonin ratios (first morning voids and overnight collection) during sessions one, two, and four. However, the correlations were very weak (*r* < 0.35) and do not currently support clinical application. To our knowledge, no other studies observed a significant correlation between caffeine and endogenous melatonin. Ursing et al.^50^ reported, for instance, a nonsignificant correlation between caffeine clearance and AUC of melatonin in 12 healthy volunteers (*r* = −0.021, *p* = 0.95). However, when measuring endogenous metrics, it seems more relevant to determine either the substrate and its metabolite simultaneously, or the metabolite alone (e.g., plasma 4‐beta‐hydroxycholesterol) rather than the substrate alone, in order to assess the activity of a CYP isoenzyme.^8^

To conclude, this study successfully demonstrated that the endogenous 6‐hydroxymelatonin/melatonin urinary ratio estimated from first morning voids and overnight collection were statistically related. Therefore, the more convenient first morning micturition could be considered when further investigating the 6‐hydroxymelatonin/melatonin urinary ratio as a metric for CYP1A2 phenotyping. Although this endogenous metabolite/substrate couple remains a potential candidate for CYP1A2 phenotyping, at present, insufficient clinical data are available to use the 6‐hydroxymelatonin/melatonin urinary ratio as a CYP1A2 metric in clinical practice, and the results of this study should be interpreted with caution. Some of the results of the present study (i.e., the larger intra‐ and interindividual variability observed in the 6‐hydroxymelatonin/melatonin ratios compared with the paraxanthine/caffeine ratio), question whether this endogenous metric adequately reflects CYP1A2 activity. Confirmatory studies with larger samples are needed in order to further explore this metric in the context of personalized medicine to measure the CYP1A2 metabolic capability of a given patient. An important validation criteria to consider when assessing new CYP450 markers is the capacity of the investigated metric (e.g., the 6‐hydroxymelatonin/melatonin ratio) to reflect modulation of the enzymatic activity after pretreatment with CYP1A2 inhibitors and/or inducers.^51^ It also seems fundamental to understand the reasons why caffeine intake had a significant impact on the paraxanthine/caffeine ratio, but no effect on the 6‐hydroxymelatonin/melatonin ratios. Finally, it may also be interesting to test the formation clearance of 6‐hydroxymelatonin, which could be a valuable metric eliminating any potential influence of the renal clearance of melatonin.^8^

## CONFLICT OF INTEREST

The authors declared no competing interests for this work.

## AUTHOR CONTRIBUTIONS

G.M. wrote the manuscript. G.M., J.D., and Y.D. designed and performed the research. G.M. and Y.D. analyzed the data. J.D., C.F.S., A.T., and Y.D. contributed new reagents/analytical tools.

## Supporting information


Appendix S1
