# Supplementary material for: Evaluation of CYP1A2 activity: Relationship between the endogenous urinary 6‐hydroxymelatonin to melatonin ratio and paraxanthine to caffeine ratio in dried blood spots
Source: Clin Transl Sci. 2022 Mar 26;15(6):1482–91. doi: 10.1111/cts.13263 (PMC9199893; doi:10.1111/cts.13263)
Supplement: Supplementary file 1 — Appendix S1 [file CTS-15-1482-s001.docx]

**SUPPLEMENTARY INFORMATION**

**Figure S1**. Paraxanthine/caffeine ratios measured in dried blood spot (DBS) samples following caffeine abstinence (session 1, 2 and 4) and following caffeine consumption (session 3). ****: *P* < 0.0001.

**Table S1**. Mean ± SD and CV% of the endogenous 6-hydroxymelatonin/melatonin ratios measured in first morning voids and 12-hour (overnight) urine samples, as well as the paraxanthine/caffeine ratio measured in dried blood spot samples at 2 hours from different sessions (sessions 1, 2, 3 and 4)

|  | *6-hydroxymelatonin/*  *melatonin (first morning voids)* | | *6-hydroxymelatonin/*  *melatonin (overnight samples)* | | *Paraxanthine/caffeine*  *(DBS at 2 hours)* | |
| --- | --- | --- | --- | --- | --- | --- |
|  | *Mean ± SD* | *CV (%)* | *Mean ± SD* | *CV (%)* | *Mean ± SD* | *CV (%)* |
| Session 1 | 133 ± 50.5 | 37.9 | 131 ± 64.8 | 49.4 | 0.22 ± 0.07 | 34.0 |
| Session 2 | 128 ± 49.0 | 38.2 | 138 ± 51.4 | 37.2 | 0.21 ± 0.05 | 23.3 |
| Session 3 | 136 ± 40.3 | 29.6 | 135 ± 42.3 | 31.3 | 0.36 ± 0.06 | 16.6 |
| Session 4 | 151 ± 87.8 | 58.3 | 155 ± 89.3 | 57.6 | 0.22 ± 0.07 | 32.9 |
| Mean ± SD |  | 41.0 ± 12.2 |  | 43.9 ± 11.9 |  | 26.7 ± 8.3 |
